# The Mediation Role of Health Behaviors in the Association between Self-Regulation and Weight Status among Preschool Children: A Sex-Specific Analysis

**DOI:** 10.3390/nu14091692

**Published:** 2022-04-19

**Authors:** Ke Xu, Yuanyuan Zhang, Wenli Dong, Paiziyeti Tuerxun, Chunan Li, Ruixia Chang, Haiqin Qi, Ya Zhang, Jianduan Zhang

**Affiliations:** Department of Maternal and Child Health, School of Public Health, Tongji Medical College, Huazhong University of Science and Technology, 13 Hangkong Rd., Wuhan 430030, China; xuke_huster@126.com (K.X.); zhyy_d@hust.edu.cn (Y.Z.); dongwenli@hust.edu.cn (W.D.); pzyt428@163.com (P.T.); lichunan95@163.com (C.L.); 13919271364@163.com (R.C.); qhq2390900032@163.com (H.Q.); 15754307651@163.com (Y.Z.)

**Keywords:** obesity, children, health behaviors, self-regulation, impulsivity

## Abstract

Previous studies have supported the link between children’s self-regulation (CSR) and weight status, but the potential pathways have not been elucidated yet. We aimed to investigate whether and to what extent health behaviors mediate this association, as well as to explore the sex effect. For this study, we recruited 3740 preschoolers in Wuhan, China. The height and weight of children were measured, and a body mass index of the ≥85th percentile was defined as overweight/obesity (OWO). We used the Children’s Behavior Questionnaire, with measured domains including inhibitory control, impulsivity, anger, and attentional focusing, to assess CSR. The primary caregivers’ SR (PSR) was assessed with the Self-Control Scale. Information on lifestyles collected from questionnaires was utilized to construct the health behavior index (HBI). We found that Children’s HBI was associated with both CSR and PSR, inhibitory control (OR = 0.81, *p* < 0.001), anger (OR = 1.23, *p* < 0.001), attentional focusing (OR = 0.70, *p* < 0.001), impulsivity (OR = 1.23, *p* < 0.001), and PSR (OR = 0.73, *p* < 0.001). Children’s impulsivity was associated with their OWO (OR = 1.11, *p* = 0.013) which was partly mediated by the HBI (direct effect: β = 0.092, *p* = 0.026; indirect effect: β = 0.011, *p* = 0.007). The sex-specific analysis indicated that this mediation effect was only significant in boys. These results indicated that impulsivity is associated with childhood weight status, which is partially mediated by health behaviors, especially in boys.

## 1. Introduction

Childhood overweight and obesity have become a public health hazard worldwide [1]. In China, the prevalence of overweight and obesity for urban children under seven years old showed a rapidly increasing trend over the last decade, reaching 8.4% in 2016 [2]. Childhood obesity could alter the life-long health trajectory, causing common chronic diseases and psychological illnesses [3,4,5]. Therefore, understanding the early related factors is of significant importance in order to prevent obesity. Unhealthy dietary patterns, insufficient physical activity, increased sedentary time, and short sleep duration are well-established risk factors for obesity. However, few obesity interventions for young children targeting health behavior changes have yielded significant improvements in weight-based outcomes, and fewer of them have reported any long-term effect [6,7]. Self-regulation (SR), the act of managing cognition and emotion, serves a fundamental role in promoting wellbeing during the course of life, with impacts on, for instance, health outcomes, emotional wellbeing, academic achievement, and socioeconomic success [8,9,10]. Emerging evidence has shown that poor children’s SR (CSR) in early childhood is associated with the risk of future obesity [10,11,12]. For example, the UK Millennium Cohort Study showed that poorer emotional CSR at age three was an independent predictor of obesity at age 11 [11]. Schlam and colleagues found that delay of gratification at preschool age, an indicator of behavioral SR, was linked with body mass index (BMI) three decades later [10]. A meta-analysis concluded that impulsivity was correlated with child obesity risk with a moderate effect size [12]. Since CSR could be altered by early intervention and produce multiple benefits beyond obesity control, enhancing CSR has been gradually regarded as a novel childhood obesity prevention measure [9,13].

While previous studies have preliminarily supported the link between CSR and weight status, few studies to date have verified this hypothesis among Chinese children and adolescents. Furthermore, the potential pathway between CSR and weight status has not been elucidated yet. Some studies have proposed that health behavior (HB), such as diet, sedentary behavior, and sleep habits, might mediate the relationship between neurocognitive functioning and obesity [14,15]. For example, Van den Berg et al. found that overeating mediated the association between impulsivity and higher BMI in children aged 6–13 years [16]. The adult research identified the mediational roles of physical activity and overeating in the association between cognitive performance and central adiposity [17,18]. However, these findings have not been replicated in some other studies. For example, in a study among preschool-aged children, Levitan et al. did not observe a mediation effect of food intake on the link between BMI and inhibitory control [19]. Overall, most related studies have targeted school-aged children and adults. Early childhood is regarded as a critical stage for neurocognitive development and obesity prevention [2,20], but few researchers have investigated the potential pathway of the CSR–obesity association in this period. In addition, mounting evidence has suggested that CSR could affect multiple obesity-related behaviors, e.g., food intake, physical activity, and sleep duration [8,15]. However, previous studies often used a single variable of health behaviors as the mediator [21,22,23], which could capture limited aspects of lifestyle. Therefore, a comprehensive index accommodating multiple energy-balanced behaviors with the weights of each behavior considered would be more feasible for assessing the HB’s role in the association between CSR and overweight/obesity (OWO).

Sex differences have been observed in neurocognitive development and weight status in young children [2,24]. Studies have shown that preschool-aged girls tend to have better self-regulation and healthier weight condition than their boy counterparts [2,24,25]. Therefore, it is worth investigating whether there is a sex disparity in the link between CSR and obesity. Emerging studies have focused on this difference in the association of obesity with inhibitory control. For example, the Maternal Adversity, Vulnerability and Neurodevelopment (MAVAN) project revealed the association between inhibitory control and higher BMIs in preschool-aged girls but not in preschool-aged boys [19], and a similar phenomenon has been observed in adolescence as well [26]. However, whether the sex disparity exists in the correlation between weight status and other dimensions of CSR has rarely been explored. Therefore, sex-specific associations and potential pathways of CSR–obesity must be further explored among different populations with larger sample sizes. In this study, we examined whether SR is associated with the weight status and lifestyle of preschoolers and, if any, to what extent the lifestyle mediated the association of SR with obesity. We also investigated whether this hypothesized association and mediation varied by sex.

## 2. Materials and Methods

### 2.1. Study Population

**Figure 1:**
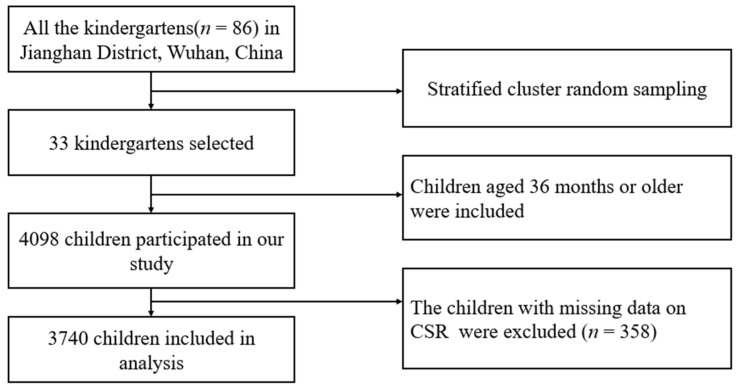
Flowchart for the current study.

We used a cross-sectional design with stratified cluster random sampling. The target population was preschool-aged children in Jianghan District, Wuhan, China, and data were collected from October to November 2020. The list of all kindergartens in the district served as the sampling frame, and each cluster indicated a kindergarten. We defined strata based on the rating of kindergartens in the official school system, i.e., provincial, municipal, and private kindergartens, and the subject kindergartens were selected with a probability proportionate to the size of each stratum. We sampled 33 of 86 kindergartens, with children aged 36 months or older eligible for our study. Written informed consent was obtained from the caregivers before data collection. Those with missing data on CSR (*n* = 358) were excluded from the analysis. Finally, 3740 children were included in our research (Figure 1).

The study was approved by the Ethics Committee of Tongji Medical College, Huazhong University of Science and Technology (Project identification code: (2020) IEC (A179)).

### 2.2. Measures

#### 2.2.1. Child Anthropometrics

The height and weight of children were measured with an automatic anthropometer (ws-rt-2u, Kangwa, China) in light clothing and barefoot. Children’s weight status was defined according to the 2006 World Health Organization (WHO) Growth Standards [27], with BMI (weight in kilograms divided by height in meters squared) within the 85–95th percentiles for age and sex as overweight, the ≥95th percentiles as obese, and the ≥85th percentiles as overweight/obese (OWO).

#### 2.2.2. Self-Regulation Measurement

The Children’s Behavior Questionnaire (CBQ) is a well-accepted assessment tool for evaluating behavior in children aged 3–8 years based on caregivers’ reports [28,29]. The validated Chinese version possesses a satisfactory internal consistency [30]. Some subscales of the CBQ that primarily reflect behavioral regulation and cognitive–emotional regulation have been widely used to measure CSR, including inhibitory control (e.g., ”Can wait before entering into new activities if s/he is asked to”), impulsivity (e.g., “Usually rushes into an activity without thinking about it”), anger (e.g., ”Gets angry when called in from play before s/he is ready to quit”), and attentional focusing (e.g., “When drawing or coloring in a book, shows strong concentration”) [8]. In our study, the primary caregivers of the children were asked to complete these subscales of the short-form CBQ by rating their child’s behaviors on a 7-point scale, i.e., from 1 (extremely untrue of your child) to 7 (extremely true of your child). An option of NA (not applicable) was provided in case the described situation had not presented in the child. Each subscale of the short-form CBQ contains 6 items. Average scores of applicable items are used as subscale scores. According to Corrected-Item Total Correlation (CITC) and Alpha Item Deleted (ID α), three items in the impulsivity subscale were removed due to poor correlation with the scale score [31]. Finally, the internal reliability Cronbach α of these subscales was 0.61–0.73.

The Self-Control Scale (SCS), a Chinese validated tool for adults’ self-regulation assessment (36 items), assesses how well adults control impulses, alter moods or emotions, restrain bad habits, maintain self-discipline, and manage performance [32]. The SCS was used to evaluate the primary caregivers’ SR (PSR) in our study [32]. Total scores were calculated, and a higher score indicates a better SR. The internal reliability was high (Cronbach α: 0.85).

### 2.3. Healthy Behaviors and Other Covariates

To reflect children’s overall health behaviors related to energy balance, we constructed a health behavior index (HBI), including diet, media use, and sleep. Dietary information was collected using food frequency questionnaires completed by caregivers to assess the food consumption frequency (i.e., daily, 3–5 times per week, 1–2 times per week, 1 time per two weeks, and occasional or no consumption) of children. The Chinese Dietary Guideline for Preschool Children recommends that children of preschool age consume vegetable and fruit every day [33]. The guideline also suggests that the children should not or only occasionally intake high-energy-density food such as fried food, fast food, dessert, puffed food, sugar-sweetened beverages, and pastry. We defined a healthy diet as meeting at least five of the recommendations from this guideline (Table S1). The average daily screen time, time to go to bed, time to get out of bed, and nap duration of the children during the past month were reported by their caregivers through the structured questionnaire. A daily screen time of less than one hour was considered to be healthy media use and a sleep duration of >10 h and a bedtime earlier than 22:30 were considered to comprise a healthy sleep status according to Physical Activity Guidelines for Chinese Preschoolers aged 3–6 years [34]. For each healthy behavior, we assigned 1 point for those defined as healthy and 0 points for those defined as unhealthy. The sum of the points was the HBI score, which ranged between 0 and 3. A higher score indicates a healthier lifestyle. Since healthy behaviors might not equally contribute to overweight/obesity, a weighted healthy behavior index (WHBI) was developed using the beta-value of each healthy behavior in the regression model for OWO as the weights (WHBI = Diet*0.22 + Sleep*0.155 + Screen time*0.350). We also collected data on physical activity (PA) through a structured questionnaire. A lack of PA is generally regarded as a risk behavior that leads to obesity. However, an association between PA and OWO has not been observed in young children in many studies [35,36], including ours. Thus, we decided not to include PA in developing HBI/WHBI. Nonetheless, as one of the common risk factors of obesity, PA was also included in the HBI in sensitivity analysis, with more than one hour of moderate-to-high-intensity physical activity per day considered to be healthy.

Other covariates included child’s sex, age, birth weight-for-length Z score, ever breastfeeding (yes or no), maternal secondhand smoke during pregnancy (never, occasional, or often), maternal current BMI, educational level (high school or less, college/university, or postgraduate or above), and household income (≤10,000, 10,001–20,000, 20,001–40,000, or ≥40,000 RMB per month).

## 3. Statistical Analysis

The characteristics of participants were described by the weight status of children and the group differences were examined using the *t* test, the χ2 test, and the Mann–Whitney U test. The scores of the CBQ and SCS were transformed into standardized scores before analysis. Logistic regression models were used to estimate the odds ratios and 95% CIs of the HBI (transformed into categorical variables: ≤2 vs. >2) after adjusting for sex, age, current maternal BMI, maternal education, and household income. Logistic regression models were also used to estimate the odds ratios and 95% CIs of OWO after adjusting for sex, age, birth weight-for-length Z scores, current maternal BMI, maternal education, household income, and secondhand smoke during pregnancy. Additionally, we applied the mediation method [37,38] based on the counterfactual framework to assess the role and the magnitude of the HBI in the association between SR and OWO (R package Medflex). The estimated total effect, natural indirect effects (NIE), and natural direct effects (NDE) were presented. The natural indirect effect (NIE) describes the idea that when controlling impulsivity at the level as naturally observed at any given level of the HBI, a change in risk of OWO is attributable to altering the level of the HBI (≤2 vs. >2) from healthy to unhealthy; the residual natural direct effect (NDE) quantifies the effect not mediated by these HBI levels. We calculated the proportion of the effect mediated by the HBI as the percentage of the natural indirect effect over the total effect. We also separately estimated the independent mediating effects of subcomponents of the HBI, i.e., diet, screen time, and sleep. Considering sexual disparities in obesity risk and child neurocognitive development, we ran a sex-stratified analysis on SR–OWO links and the mediations effect of the HBI. Missing values of the covariates were filled in by multiple imputations using the R package mice.

Several sensitivity analyses were performed. First, we excluded children whose questionnaires were not completed by their parents (*n* = 230). Second, we restricted the analysis to those children without prevalent diabetes, thyroid diseases, or pituitary disorders (*n* = 49). Third, we applied the following variables as mediators: the HBI included the physical activity component and the WHBI.

All analyses were performed using R (3.6.0). All statistical tests were 2-sided, with the statistical significance set at 0.05.

## 4. Results

### 4.1. Population Characteristics

Of the 3740 children included analysis, 2019 (54.0%) were boys, the mean (SD) age was 55.65 (10.26) months, and the mean (SD) birth weight-for-length Z score was −0.19 (3.01). Over half children were ever breastfed (*n* = 3121, 83.4%). Most of their mothers had a college education (*n* = 2424, 64.8%), and over half of their household incomes were lower than 20,000 RMB per month (*n* = 2352, 62.9%). The prevalence of OWO was 21.17% (792), 11.0% (412) for overweight and 10.16% (380) for obesity. Table 1 shows sociodemographic characteristics of the overweight/obesity (OWO) and comparator groups. OWO children were more likely to be boys, of an older age, and have a higher maternal BMI. The mean (SD) values of the HBI and WHBI were 1.77 (0.95) and 0.49 (0.24), respectively. The mean scores of CSR domains were: inhibitory control (5.09, SD: 0.91), impulsivity (3.88, SD: 1.07), anger (4.10, SD: 0.93), and attentional focusing (4.91, SD: 0.93), and the mean PSR score was 73.00 (72.83, SD: 8.84). In addition, girls processed significantly higher scores in inhibitory control (*p* < 0.001) and attentional focusing (*p* < 0.001) than boys, whereas the result was the opposite for impulsivity (*p* = 0.002).

### 4.2. Association between SR, Health Behavior and Obesity

**Figure 2:**
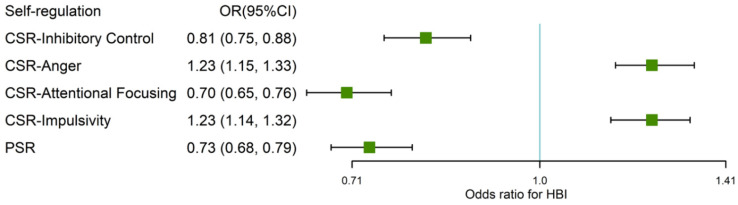
ORs and 95% CIs for the association of SR–HBI. Logistic regression models were used to calculate ORs and 95% CIs. All the models were adjusted children sex, age, current maternal BMI, maternal education, and household income. OR: odds ratio; HBI: health behavior index; CSR: children’s self-regulation; PSR: primary caregivers’ self-regulation. HBI values (≤ 2 vs. >2) were transformed into categorical variables.

As shown in Figure 2, both CSR and PSR were associated with children’ HBI after adjusting sex, age, current maternal BMI, maternal education, and household income. One standard deviation (SD) increments in the score of inhibitory control, attentional focusing, and PSR were associated with 19% (OR = 0.81; 95% CI = 0.75–0.88), 30% (OR = 0.70; 95% CI = 0.65–0.76), and 27% (OR = 0.73; 95% CI = 0.68–0.79) average reductions in the odds of having a poorer HBI (HBI ≤ 2), respectively. Additionally, one SD increment in the score of anger was associated with a 23% increment (OR = 1.23; 95% CI = 1.15–1.33) in the odds of a poorer HBI (HBI ≤ 2); the figure for impulsivity was also 23% (OR = 1.23; 95% CI = 1.14–1.32). No clear differences were observed when the associations of CSR and PSR with the HBI were analyzed in groups stratified by children’s sex (Table S2).

**Figure 3:**
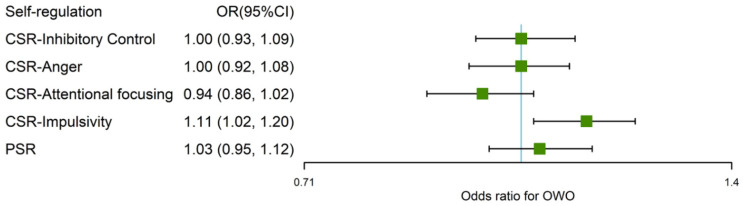
ORs and 95% CIs for the association of SR–OWO. Logistic regression models were used to calculate ORs and 95% CIs. All the models were adjusted sex, age, birth weight-for-length Z scores, current maternal BMI, maternal education, household income, and secondhand smoke during pregnancy. OR: odds ratio; OWO: overweight/obesity; CSR: children’s self-regulation; PSR: primary caregivers’ self-regulation.

As shown in Figure 3, we found that only impulsivity was statistically associated with OWO after adjusting for sex, age, birth weight-for-length Z scores, current maternal BMI, maternal education, household income, and secondhand smoke during pregnancy. One SD increment in the score of impulsivity was associated with an 11% average increment (OR = 1.11; 95% CI = 1.02–1.20) in the odds of OWO. The significant correlation between impulsivity and OWO was only observed in boys (OR = 1.13; 95% CI = 1.02–1.25) but not in girls (OR = 1.07; 95% CI = 0.94–1.23) (Table S3). Tests for multicollinearity indicated that no significant multicollinearity was found (variance inflation factor: ≤1.5) for all variables.

### 4.3. Mediation Analysis of HBI on Associations of Impulsivity with OWO

Considering the significant correlation between impulsivity and OWO, mediation analysis was further conducted to test the mediation effect of the HBI. As presented in Table 2**,** the HBI partly mediated the impulsivity–obesity association (direct effect: β = 0.092, *p* = 0.026; indirect effect: β = 0.011, *p* = 0.007). The indirect association via the HBI implied that when controlling impulsivity at the level naturally observed at any given level of the HBI, altering the level of the HBI from healthy to unhealthy would lead to an exponential (0.011)-fold increased risk of OWO. A similar medication effect only emerged in boys (direct effect: β = 0.109, *p* = 0.044; indirect effect: β = 0.010, *p* = 0.032).

We further conducted mediation analysis using the health behavior subcomponents, including diet, sleep status, and screen time, as the mediators (Table 2). All the subcomponents except sleep partly mediated the impulsivity–obesity association. The subcomponent that accounted for the largest mediation proportion was screen time (9.71%), followed by diet (5.83%). Among boys, the indirect associations for each health behavior subcomponent were insignificant. None of the total, direct, or indirect associations remained significant among girls.

### 4.4. Sensitivity Analysis

The results of the sensitivity analysis—in which excluded children whose questionnaires were not completed by their parents or those with prevalent diabetes, thyroid diseases, or pituitary disorders—were similar to the main analysis. No variation in mediation analysis emerged when the HBI included the physical activity component or used the WHBI (Table S4).

## 5. Discussion

In this large sample of Chinses preschoolers, we found that both CSR and PSR were associated with children’s HBI. Impulsivity was identified as a risk factor for childhood weight status, and the HBI partly mediated the impulsivity–obesity association. In sex-specific analysis, the HBI’s mediation effect was only significant among boys. With regard to individual components of health behavior, it seems that screen time mediated the highest proportion of association between impulsivity and obesity, followed by diet.

Similar to other research [21,22,23], children who tend to act impulsively and easily become angry were found to be more likely to have a poorer HBI, while those with higher inhibitory control and attentional focusing tended to have a better HBI. There are several possible mechanisms behind these results. For example, obesity-associated impulsivity and the increased consumption of high-calorie food share a common neural mechanism, i.e., reduced medial prefrontal cortical (PFC) brain function [39]. Likewise, the dorsolateral PFC is considered to be the neuroanatomical basis for inhibitory control, and it plays an important role in hedonic feeding inhibition [40]. This finding suggests that these facets of CSR could have vital impacts on overall health behaviors, reinforcing the value of interventions to promote CSR. One of the novel findings in our study was that children whose caregivers had greater SR scores were inclined to adopt healthier behaviors. Previous studies on adults have reported that those with lower levels of SR engaged in less consumption of fruit and vegetable and shorter average exercise sessions [15,41,42]. We assumed that on the one hand, a greater PSR could help caregivers to better govern their children’s food choices and physical activity on a daily basis, and on the other hand, caregivers with better SR scores could demonstrate an improved HB for their children as a positive role model. This finding indicates that interventions with the component of strengthening caregivers’ awareness of the importance of PSR and improving PSR levels could be beneficial to the development of health-enhancing habits in children.

Impulsivity, an indicator of poor behavioral SR, has been linked to several high-risk behaviors, such as substance use, gambling, and binge eating [43,44]. Our results evidenced that impulsivity is associated with a higher risk of obesity in preschoolers. This is consistent with a meta-analysis of school-aged children and adolescents that showed that impulsivity plays an essential role in obesity with a moderate effect size [12]. Bidirectional causality between impulsivity, inattention symptoms, and obesity-related traits among adolescent was identified by a Mendelian randomization analysis. Possible mechanisms might include the genetic overlap between impulsivity symptoms and obesity-related traits and the abnormal functioning of the dopamine pathway modulating the consumption of food [45]. However, not all research has detected this association, probably due to variable measurement of impulsivity, different cultural contexts, and different ages of subjects [46]. As for the other dimensions of CSR, we did not detect their impact on children’s adiposity, probably due to different measurements of neurocognition and cross-sectional design.

Prior studies have solely focused on the main effect of CSR on obesity and have overlooked the possibility of multiple pathways and processes. The current study confirmed the mediator role of overall health behaviors in the association of impulsivity and obesity. Some research on school-aged children and adolescents has revealed indirect effects through eating behaviors in the CSR–adiposity association [47,48]. To our knowledge, few studies have estimated the mediation effect of factors other than dietary behavior. Our findings add new evidence regarding the mediation effect of overall lifestyle from preschooler populations, strengthening the value of incorporating CSR component into childhood obesity intervention programs targeting lifestyle. There are several possible explanations for this mediated effect. For example, according to dual-process models, children with poor SR scores tend to show heightened responsivity to high-energy-density food (strong automatic processing) and/or weak self-control (regulatory processing), resulting in more snaking or excessive weight gain [49]. As for the subcomponents of the HBI, diet and screen time were identified as the independent mediators in our study. This suggests that, in addition to eating behavior, the pathways between CSR and OWO might involve the change of sedentary behavior in preschoolers, which has seldom been reported before. The association of CSR with OWO did not appear to be mediated by sleep status, probably due to the limited impact of sleep status on OWO in our study. Further study is required to confirm this conclusion.

We found sex-specific differences in the CSR distribution, impulsivity–OWO association, and mediation effect of the HBI. The current study revealed that boys were more likely to be impulsive, inattentive, and have a lower inhibitory control than the girls, consistent with previous investigations [24,25]. Moreover, we only identified a significant impulsivity–OWO association in boys, which has been rarely reported. However, our result did not support the conclusion of previous studies that sex discrepancy exists in the association between inhibitory control and the BMI [19,26], probably due to variable measurements, different cultural contexts, and different ages of subjects. Another possible interpretation is that inhibitory control has an effect on subsequent adiposity that emerges early in girls [26], and it might not be identifiable in a cross-sectional study. Further research is needed to confirm the sex differences in other dimensions of SR and whether these discrepancies exists in periods other than early childhood. Probably due to the nonsignificant association of impulsivity–OWO among girls, we did not identify a mediated effect of the HBI in this subgroup. Our results indicated that preschooler boys, especially those with high levels of impulsivity, probably received multiple benefits from interventions with activities designed to promote CSR. Future research with longitudinal data needs to replicate our findings and detect underlying the biological mechanisms of the sex differences.

## 6. Strengths and Limitations

In addition to the relatively large sample size, our research constructed the HBI and WHBI to comprehensively reflect the overall energy-balance lifestyle. These indexes allowed us to identify the mediator role of comprehensive HB in the CSR–obesity association, rather than any of the single health behaviors. Moreover, PSR, a factor often overlooked by previous studies, was considered to assess the impact on offspring’s health outcomes, resulting in new evidence for the important role of PSR on children’s health-prompting. We also found a sex difference in the link between impulsivity and weight status and the mediation effect, which bolstered the necessity of developing targeted interventions for early obesity prevention and control.

We also acknowledge several limitations in our study. First, the cross-sectional nature of our study does not permit causal inferences, e.g., it could not determine whether poor CSR results from worse health behaviors or whether better CSR facilitates health behaviors. Second, the CSR and HB of children were reported by caregivers, which could have been less objective than direct behavior assessments and led to measurement errors. For CSR, the CBQ is a common measurement with good reliability and validity.

## 7. Conclusions

In our study, impulsivity was associated with childhood weight status, and the HBI partly mediated the impulsivity–OWO association. Moreover, this mediation effect only emerged among boys. Our result suggests that promoting health behaviors alone without considering SR might not be effective in childhood obesity prevention. Longitudinal studies need to replicate this finding and understand the cause of the sex-specific differences in mediation analysis.

## Figures and Tables

**Table 1:** Sociodemographic characteristics of the children ^a^.

Variables	Total Population (*n* = 3740)	Normal Weight (*n* = 2948)	Overweight/Obesity (*n* = 792)	*p*-Value
Mean age (mean (SD), months)	55.65 (10.26)	55.25 (10.30)	57.16 (9.94)	<0.01 **
Age *n* (%)				<0.01 **
36~48 months	950 (25.4)	802 (27.2)	148 (18.7)	
48~60 months	1428 (38.2)	1109 (37.6)	319 (40.3)	
≥60 months	1362 (36.4)	1037 (35.2)	325 (41.0)	
Sex *n* (%)				<0.01 **
Boy	2019 (54.0)	1503 (51.0)	516 (65.2)	
Girl	1721 (46.0)	1445 (49.0)	276 (34.8)	
Birth weight-for-length Z scores (mean (SD)) ^b,c^	−0.19 (3.01)	−0.22 (3.02)	−0.09 (2.95)	0.300
Ever breastfeeding *n* (%) ^c^				0.278
No	453 (12.1)	362 (12.3)	91 (11.5)	
Yes	3121 (83.4)	2463 (83.5)	658 (83.1)	
Current Maternal BMI (median (IQR), kg/m^2^) ^c^	20.83 (19.43, 22.77)	20.66 (19.23, 22.49)	21.83 (20.06, 23.88)	<0.01 **
Maternal education ^c^				0.896
High school or less	794 (21.2)	623 (21.1)	171 (21.6)	
College/University	2424 (64.8)	1907 (64.7)	517 (65.3)	
Postgraduate or above	404 (10.8)	323 (11.0)	81 (10.2)	
Household income (*n* (%), RMB per month) ^c^				0.388
≤10,000	1014 (27.1)	810 (27.5)	204 (25.8)	
10,001~20,000	1338 (35.8)	1031 (35.0)	307 (38.8)	
20,001~40,000	910 (24.3)	724 (24.6)	186 (23.5)	
≥40,001	293 (7.8)	233 (7.9)	60 (7.6)	
Secondhand smoke during pregnancy *n* (%) ^c^				0.970
Never	2640 (70.6)	2081 (70.6)	559 (70.6)	
Occasional	1013 (27.1)	800 (27.1)	213 (26.9)	
Often	59 (1.6)	45 (1.5)	14 (1.8)	

^a^. *p*-values were calculated using the analysis of the *t* test, Mann–Whitney U test and the χ2 test. ^b^. Birth weight-for-length Z scores: birth weight and length were used to calculated Z scores based on WHO child growth standards. ^c^. Number of participants with missing information: Birth weight-for-length Z scores (386), ever breastfeeding (166), current Maternal BMI (98), maternal education (118), household income (185), and secondhand smoke during pregnancy (28). ** *p* < 0.01.

**Table 2:** Results of mediation analysis between impulsivity and weight status explained by the HBI and its subcomponents ^a^.

	Overall (*n* = 3740)		Boys (*n* = 2019)		Girls (*n* = 1721)	
	β	*p*	β	*p*	β	*p*
Mediator: HBI ^a^						
Direct effects	0.092	0.026 *	0.109	0.044 *	0.060	0.364
Indirect effects	0.011	0.007 **	0.010	0.032 *	0.011	0.124
Total effect	0.103	0.013 *	0.119	0.027 *	0.071	0.277
Proportion mediated, %	10.67		8.40		15.49	
Mediator: Diet ^b^						
Direct effects	0.097	0.020 *	0.114	0.035 *	0.063	0.340
Indirect effects	0.006	0.043 *	0.005	0.155	0.008	0.126
Total effect	0.103	0.013 *	0.119	0.028 *	0.071	0.277
Proportion mediated, %	5.83		4.20		11.26	
Mediator: Screen time ^c^						
Direct effects	0.093	0.025 *	0.111	0.040 *	0.058	0.374
Indirect effects	0.010	0.010 *	0.008	0.090	0.013	0.054
Total effect	0.103	0.013 *	0.119	0.027 *	0.071	0.277
Proportion mediated, %	9.71		6.72		18.31	
Mediator: Sleep ^d^						
Direct effects	0.100	0.017 *	0.118	0.029 *	0.061	0.346
Indirect effects	0.003	0.148	0.001	0.553	0.010	0.118
Total effect	0.103	0.013 *	0.119	0.027 *	0.071	0.277
Proportion mediated, %	2.91		0.84		14.08	

HBI: Health behavior index. ^a^. HBI values were transformed into categorical variables (≤2 vs. >2). Model adjusted children’s sex, age, birth weight-for-length Z scores, current maternal BMI, maternal education, household income, and secondhand smoke during pregnancy; ^b^. Diet: categorical variables. Meeting at least 5 items of the dietary recommendations was considered to reach the healthy level; ^c^. Screen time: categorical variables; <1 h/day was considered a healthy level; ^d^. Sleep: categorical variables; ≥10 h/day (bedtime before 22:30) was considered a healthy level. * *p* < 0.05, ** *p* < 0.01.

## Data Availability

The data are not publicly available due to privacy.

## Supplementary Materials

The following supporting information can be downloaded at: https://www.mdpi.com/article/10.3390/nu14091692/s1, Table S1: Components of healthy behaviors. Table S2: Associations of HBI with self-regulation of children and primary caregivers ^a^. Table S3: Associations of OWO with self-regulation of children and primary caregivers ^a^. Table S4: The association between children overweight/obesity and CSR and PSR, as well as the mediating role of HB: sensitivity analyses.
