# Supplementary material for: The Mediation Role of Health Behaviors in the Association between Self-Regulation and Weight Status among Preschool Children: A Sex-Specific Analysis
Source: Nutrients. 2022 Apr 19;14(9):1692. doi: 10.3390/nu14091692 (PMC9104780; doi:10.3390/nu14091692)
Supplement: Supplementary file 1 [file nutrients-14-01692-s001.zip › nutrients-1655417-supplementary.pdf]

Table S1. Components of healthy behaviors.

	Definition	*n* (%)
Diet [a]		
Vegetable	Everyday	3015 (80.6)
Fruit	Everyday	2609 (69.8)
Fried food	Occasional or no consumption	2024 (54.1)
Fast-food	Occasional or no consumption	2713 (72.5)
Dessert	Occasional or no consumption	2012 (53.8)
Puffed food	Occasional or no consumption	2357 (63.0)
Sugar-sweetened beverages	Occasional or no consumption	3008 (80.4)
Pastry	Occasional or no consumption	603 (16.1)
Healthy diet [b]		2162 (63.2)
Unhealthy diet		1258 (36.8)
Screen time	<1 h/day	1885 (50.4)
Sleep duration	≥10 h/day (bedtime before 22:30)	2024 (54.1)

[a]. Based on children's food frequency in the past three months [b]. A healthy diet was defined as meeting at least five of the dietary recommendations.

Table S2. Associations of HBI with self-regulation of children and primary caregivers [a].

	Model1 [b]		Model2 [c]	
	OR (95% CI)	*p*	OR (95% CI)	*p*
Overall				
CSR–Inhibitory control	0.81 (0.75, 0.87)	<0.001 **	0.81 (0.75, 0.88)	<0.001 **
CSR–Anger	1.24 (1.15, 1.33)	<0.001 **	1.23 (1.15, 1.33)	<0.001 **
CSR–Attentional focusing	0.70 (0.65, 0.76)	<0.001 **	0.70 (0.65, 0.76)	<0.001 **
CSR–Impulsivity	1.23 (1.14, 1.32)	<0.001 **	1.23 (1.14, 1.32)	<0.001 **
PSR	0.74 (0.68, 0.80)	<0.001 **	0.73 (0.68, 0.79)	<0.001 **
Boy				
CSR–Inhibitory control	0.81 (0.73, 0.90)	<0.001 **	0.82 (0.74, 0.91)	<0.001 **
CSR–Anger	1.29 (1.17, 1.43)	<0.001 **	1.28 (1.16, 1.42)	<0.001 **
CSR–Attentional focusing	0.66 (0.59, 0.74)	<0.001 **	0.66 (0.59, 0.74)	<0.001 **
CSR–Impulsivity	1.20 (1.08, 1.33)	<0.001 **	1.19 (1.08, 1.33)	0.001 **
PSR	0.72 (0.65, 0.80)	<0.001 **	0.71 (0.64, 0.79)	<0.001 **
Girl				
CSR–Inhibitory control	0.80 (0.72, 0.90)	<0.001 **	0.80 (0.71, 0.90)	<0.001 **
CSR–Anger	1.18 (1.07, 1.32)	0.001 *	1.18(1.06, 1.32)	0.002 *
CSR–Attentional focusing	0.75 (0.67, 0.84)	<0.001 **	0.75 (0.67, 0.84)	<0.001 **
CSR–Impulsivity	1.26 (1.13, 1.41)	<0.001 **	1.27 (1.14, 1.42)	<0.001 **
PSR	0.75 (0.68, 0.84)	<0.001 **	0.75 (0.67, 0.84)	<0.001 **

HBI: health behavior index; CSR: children' self-regulation; PSR: primary caregivers' self-regulation [a]. OR (95% CI) and *p*-values were calculated using logistics models. HBI values (≤2 vs. >2) were transformed into categorical variables. [b]. model 1 adjusted sex, age, and current maternal BMI; sex was not adjusted in sex-stratified analyses; [c]. model 2 adjusted sex, age, current

maternal BMI, maternal education, and household income; sex was not adjusted in sex-stratified analyses; * $p < 0.05$, ** $p < 0.01$.

Table S3. Associations of OWO with self-regulation of children and primary caregivers [a].

	Model1 [b]		Model2 [c]	
	**OR (95% CI)**	***p***	**OR (95% CI)**	***p***
Overall				
CSR–Inhibitory control	1.01 (0.93, 1.10)	0.737	1.00 (0.93, 1.09)	0.910
CSR–Anger	1.00 (0.92, 1.08)	0.938	1.00 (0.92, 1.08)	0.994
CSR–Attentional focusing	0.95 (0.88, 1.03)	0.209	0.94 (0.86, 1.02)	0.112
CSR–Impulsivity	1.10 (1.01, 1.19)	0.023 *	1.11 (1.02, 1.20)	0.013 *
PSR	1.04 (0.96, 1.12)	0.393	1.03 (0.95, 1.12)	0.484
Boy				
CSR–Inhibitory control	1.03 (0.93, 1.14)	0.551	1.02 (0.93, 1.13)	0.643
CSR–Anger	1.01 (0.91, 1.12)	0.882	1.01 (0.91, 1.12)	0.877
CSR–Attentional focusing	0.92 (0.83, 1.02)	0.122	0.91 (0.82, 1.01)	0.066
CSR–Impulsivity	1.12 (1.01, 1.24)	0.036 *	1.13 (1.02, 1.25)	0.024 *
PSR	1.01 (0.91, 1.12)	0.853	1.00 (0.90, 1.11)	0.949
Girl				
CSR–Inhibitory control	0.98 (0.85, 1.13)	0.775	0.97 (0.84, 1.11)	0.647
CSR–Anger	0.98 (0.86, 1.11)	0.705	0.97 (0.86, 1.11)	0.701
CSR–Attentional focusing	0.99 (0.87, 1.13)	0.909	0.98 (0.86, 1.12)	0.800
CSR–Impulsivity	1.07 (0.94, 1.22)	0.314	1.07 (0.94, 1.23)	0.297
PSR	1.08 (0.95, 1.23)	0.246	1.08 (0.95, 1.23)	0.245

OWO: overweight/obesity; CSR: children' self-regulation; PSR: primary caregivers' self-regulation [a]. OR (95% CI) and *p*-values were calculated using logistics models. [b]. model 1 adjusted sex, age, and current maternal BMI; sex was not adjusted in sex-stratified analyses; [c]. model 2 adjusted sex, age, birth weight-for-length Z scores, current maternal BMI, maternal education, household income, and secondhand smoke during pregnancy; sex was not adjusted in sex-stratified analyses; * $p < 0.05$.

Table S4. The association between children overweight/obesity and CSR and PSR, as well as the mediating role of HB: sensitivity analyses.

Analysis	Model 1 [a]	Mediation Analysis		
	OR (95% CI)	Direct Effect β	Indirect Effect β	Total Effect β
Excluding children whose questionnaires were not completed by their parents [b]				
CSR–Inhibitory control	1.00 (0.92, 1.09)			
CSR–Anger	0.99 (0.91, 1.08)			
CSR–Attentional focusing	0.93 (0.86, 1.02)			
CSR–Impulsivity	1.11 (1.02, 1.21) *	0.093 *	0.011 **	0.105 *
PSR	1.02 (0.93, 1.11)			
Excluding children with diabetes, thyroid diseases, or pituitary disorders [c]				
CSR–Inhibitory control	1.01 (0.93, 1.09)			
CSR–Anger	1.00 (0.92, 1.08)			
CSR–Attentional focusing	0.93 (0.86, 1.01)			
CSR–Impulsivity	1.11 (1.03, 1.21) *	0.098 *	0.011 **	0.109 **
PSR	1.03 (0.95, 1.12)			
Healthy behavior score including physical activity as the mediator [d,f]				
CSR–Impulsivity	-	0.093 *	0.011 **	0.103 *
Weighted Healthy behavior score as the mediator [e]				
CSR–Impulsivity	-	0.087 *	0.016 **	0.103 *

HB: healthy behavior; CSR: children' self-regulation; PSR: primary caregiver self-regulation [a]. Model 1 adjusted children sex, age, birth weight-for-length Z scores, current maternal BMI, maternal education, household income, and secondhand smoke during pregnancy; [b]. sample size: n = 3510; [c]. sample size: n = 3691; [d]. sample size: n = 3740; [e]. sample size: n = 3740; [f]. physical activity: >1 h/day (moderate to high intensity activity) was defined as healthy level; * $p < 0.05$, ** $p < 0.01$.
